# Genome-wide characterization of the rose (*Rosa chinensis*) WRKY family and role of RcWRKY41 in gray mold resistance

**DOI:** 10.1186/s12870-019-2139-6

**Published:** 2019-11-27

**Authors:** Xintong Liu, Dandan Li, Shiya Zhang, Yaling Xu, Zhao Zhang

**Affiliations:** Beijing Key Laboratory of Development and Quality Control of Ornamental Crops, Department of Ornamental Horticulture, China Agricultural University, Yuanmingyuan Xilu 2, Beijing, 100193 China

**Keywords:** *Rosa* sp., WRKY, Transcription factor, *Botrytis cinerea*, Grey mold

## Abstract

**Background:**

The WRKYs are a major family of plant transcription factors that play roles in the responses to biotic and abiotic stresses; however, a comprehensive study of the WRKY family in roses (*Rosa* sp.) has not previously been performed.

**Results:**

In the present study, we performed a genome-wide analysis of the *WRKY* genes in the rose (*Rosa chinensis*), including their phylogenetic relationships, gene structure, chromosomal locations, and collinearity. Using a phylogenetic analysis, we divided the 56 *RcWRKY* genes into three subgroups. The *RcWRKY*s were unevenly distributed across all seven rose chromosomes, and a study of their collinearity suggested that genome duplication may have played a major role in *RcWRKY* gene duplication. A Ka/Ks analysis indicated that they mainly underwent purifying selection. *Botrytis cinerea* infection induced the expression of 19 *RcWRKY*s, most of which had undergone gene duplication during evolution. These *RcWRKY*s may regulate rose resistance against *B. cinerea*. Based on our phylogenetic and expression analyses, *RcWRKY41* was identified as a candidate regulatory gene in the response to *B. cinerea* infection, which was confirmed using virus-induced gene silencing.

**Conclusions:**

This study provides useful information to facilitate the further study of the function of the rose *WRKY* gene family.

## Background

Transcription factors play crucial roles in plant growth, development, metabolism, and stress responses. Transcription factors usually possess a DNA-binding domain, a transactivation domain, an oligomerization site, and a nuclear localization signal, among other domains. The WRKYs are one of the most important transcription factor families in plants. These proteins all possess at least one WRKY domain [1], a DNA-binding domain which binds the W-box (TTGACC/T) sequence of the promoter region in their target genes to regulate their expression. In addition, the C-terminal of the WRKY transcription factors usually contains a zinc finger structure. In *Arabidopsis thaliana*, the WRKY protein family can be divided into three different groups: Group I proteins contain two WRKY domains and Group II WRKYs contain only one WRKY domain. Group III proteins also possess a single WRKY domain, but their zinc finger structure is unique from those of the other two groups [2].

WRKY transcription factors participate in the regulation of various plant processes, including growth and development, the response to abiotic stresses, and disease resistance; for example, AtWRKY45 is involved in the regulation of plant leaf senescence through the gibberellin signaling pathway [3]. In rice (*Oryza sativa*), OsWRKY53 positively regulates brassinosteroid signals to influence the plant architecture [4]. The expression of *AtWRKY22* in Arabidopsis is strongly induced by submergence during flooding, and its protein product binds to the promoter of *TREHALASE1*, which involved in stomatal function, to inhibit its expression [5]. *AtWRKY8* is highly expressed in plant roots and is significantly upregulated under salt stress, with *atwrky8* knockout mutants showing a greater sensitivity to salt [6]. The WRKYs are also key players in plant resistance responses against pathogens; for example, AtWRKY33 is activated by a MAPK signaling pathway and regulates the biosynthesis of phytoalexin to enhance pathogen resistance in Arabidopsis [7]. By contrast, *AtWRKY38* and *AtWRKY62* encode two structurally similar WRKYs that negatively regulate the defense against *Pseudomonas syringae*; the overexpression of these two genes decreased plant resistance to this pathogen, and the *atwrky38*, *atwrky62*, and *atwrky38 atwrky62* loss-of-function mutants displayed an enhanced disease resistance [8]. These results indicate that the WRKYs play both positive and negative regulatory roles in plant basal disease resistance.

Roses (*Rosa* sp.) are one of the most important commercial flower crops worldwide [9]. The major rose-producing areas include the tropical plateau regions of Africa and South America (including Kenya, Ethiopia, Ecuador, and Colombia), which have suitable climatic conditions and low labor costs, while rose purchasing is largely concentrated in developed countries in Europe and North America [10]. The long-distance logistics and transportation of roses pose a challenge for their post-harvest preservation, with flowers often being affected by post-harvest diseases such as gray mold caused by the necrotrophic fungal pathogen *Botrytis cinerea* [11].

Some WRKYs enhance the resistance of crops and model plants such as Arabidopsis against various diseases, including *B. cinerea*; however, the *WRKY* genes involved in gray mold resistance in roses have not yet been identified. We previously explored the molecular basis of rose resistance against *B. cinerea* using a de novo RNA-Seq analysis, revealing that large numbers of genes, including *WRKY* family genes, were significantly upregulated in roses upon *B. cinerea* infection [10]. In the present study, we performed a genome-wide analysis of the WRKY family in roses, and used virus-induced gene silencing (VIGS) to confirm that *RcWRKY41* plays an important role in rose resistance against gray mold.

## Results

### Identification of the *RcWRKY* genes in rose

To identify the rose *WRKY* family gene, the WRKY HMM profile (Pfam: 03106) was used as a query to search the rose genome database (*Rosa chinensis* Homozygous Genome v2.0; available at https://lipm-browsers.toulouse.inra.fr/pub/RchiOBHm-V2/) [12]. The HMM search led to the identification of 56 candidate *RcWRKY* genes in the rose genome. We examined the sequences of all candidate proteins using the Conserved Domain Database (https://www.ncbi.nlm.nih.gov/Structure/cdd/wrpsb.cgi), verifying that all 56 RcWRKY proteins contained the WRKY DNA-binding domain. A total of 25 of the candidate RcWRKY proteins contained two WRKY domains, while the other 31 contained one WRKY domain. All 56 *RcWRKY* genes could be mapped onto the rose chromosomes, and were named *RcWRKY1* to *RcWRKY56* according to their order on the chromosomes.

The sizes of the RcWRKY proteins varied dramatically. RcWRKY19 was the longest, containing 729 amino acids, while the shortest was RcWRKY10, comprising just 120 amino acids. The average length of the RcWRKY proteins was 359 amino acids. Details of the *RcWRKY* genes, including their accession numbers, chromosomal locations, number of introns and exons, protein sizes, and gene classifications, are provided in Table 1.

**Table 1:** Members of the *RcWRKY* gene family, as predicted in *R. chinensis* genome sequence

Gene	Accession number^a^	Chr.^b^	Position^c^	Intron	Exon	CDS (bp)	Amino Acids	Clade
RcWRKY1	RchiOBHm_Chr1g0348121	1	40.91	4	5	894	297	I
RcWRKY2	RchiOBHm_Chr1g0357671	1	50.01	2	3	990	329	II
RcWRKY3	RchiOBHm_Chr1g0357751	1	50.05	1	2	681	226	I
RcWRKY4	RchiOBHm_Chr1g0359091	1	51.09	2	3	1053	350	III
RcWRKY5	RchiOBHm_Chr1g0372431	1	61.12	2	3	1047	348	III
RcWRKY6	RchiOBHm_Chr1g0372521	1	61.17	2	3	501	166	III
RcWRKY7	RchiOBHm_Chr1g0378621	1	65.04	2	3	1281	426	II
RcWRKY8	RchiOBHm_Chr1g0380121	1	65.88	2	3	1101	366	III
RcWRKY9	RchiOBHm_Chr2g0106361	2	17.77	2	3	945	314	II
RcWRKY10	RchiOBHm_Chr2g0117181	2	29.47	1	2	363	120	I
RcWRKY11	RchiOBHm_Chr2g0117781	2	30.1	2	3	741	246	I
RcWRKY12	RchiOBHm_Chr2g0130891	2	46.96	2	3	741	246	II
RcWRKY13	RchiOBHm_Chr2g0133001	2	49.62	4	5	975	324	II
RcWRKY14	RchiOBHm_Chr2g0151681	2	69.24	2	3	1113	370	I
RcWRKY15	RchiOBHm_Chr2g0156771	2	73.35	4	5	1431	476	I
RcWRKY16	RchiOBHm_Chr2g0166991	2	81.67	3	4	1605	534	I
RcWRKY17	RchiOBHm_Chr2g0169011	2	83.29	3	4	1473	490	II
RcWRKY18	RchiOBHm_Chr2g0175911	2	87.91	1	2	606	201	I
RcWRKY19	RchiOBHm_Chr3g0447881	3	0.39	4	5	2190	729	I
RcWRKY20	RchiOBHm_Chr3g0450591	3	2.07	2	3	945	314	II
RcWRKY21	RchiOBHm_Chr3g0460351	3	8.49	2	3	1122	373	III
RcWRKY22	RchiOBHm_Chr3g0460361	3	8.51	2	3	987	328	III
RcWRKY23	RchiOBHm_Chr3g0461481	3	9.53	4	5	1521	506	I
RcWRKY24	RchiOBHm_Chr3g0466341	3	12.8	2	3	879	292	I
RcWRKY25	RchiOBHm_Chr3g0468221	3	14.29	6	7	1437	478	I
RcWRKY26	RchiOBHm_Chr3g0485711	3	32.49	4	5	1344	447	II
RcWRKY27	RchiOBHm_Chr3g0487201	3	34.38	2	3	963	320	I
RcWRKY28	RchiOBHm_Chr4g0398741	4	15.39	2	3	918	305	II
RcWRKY29	RchiOBHm_Chr4g0425801	4	51.41	2	3	1167	388	I
RcWRKY30	RchiOBHm_Chr4g0429851	4	54.56	1	2	576	191	I
RcWRKY31	RchiOBHm_Chr4g0438661	4	61.29	3	4	969	322	I
RcWRKY32	RchiOBHm_Chr4g0439041	4	61.49	3	4	1551	516	I
RcWRKY33	RchiOBHm_Chr4g0440391	4	62.52	2	3	1668	555	II
RcWRKY34	RchiOBHm_Chr5g0002561	5	1.49	5	6	1752	583	II
RcWRKY35	RchiOBHm_Chr5g0011581	5	7.68	2	3	834	277	II
RcWRKY36	RchiOBHm_Chr5g0013131	5	8.9	2	3	1062	353	I
RcWRKY37	RchiOBHm_Chr5g0018041	5	12.58	2	3	1491	496	II
RcWRKY38	RchiOBHm_Chr5g0040801	5	35.42	5	6	1629	542	II
RcWRKY39	RchiOBHm_Chr5g0042581	5	37.45	5	6	1746	581	I
RcWRKY40	RchiOBHm_Chr5g0042601	5	37.46	2	3	381	126	I
RcWRKY41	RchiOBHm_Chr5g0071811	5	77.65	5	6	1872	623	I
RcWRKY42	RchiOBHm_Chr5g0074411	5	80.32	6	7	2151	716	I
RcWRKY43	RchiOBHm_Chr5g0083891	5	89.56	2	3	423	140	I
RcWRKY44	RchiOBHm_Chr6g0289301	6	52.4	3	4	1053	350	II
RcWRKY45	RchiOBHm_Chr6g0299481	6	60.54	1	2	918	305	II
RcWRKY46	RchiOBHm_Chr6g0299501	6	60.55	4	5	1176	391	II
RcWRKY47	RchiOBHm_Chr6g0305101	6	64.18	2	3	1020	339	II
RcWRKY48	RchiOBHm_Chr6g0308491	6	66.57	2	3	969	322	II
RcWRKY49	RchiOBHm_Chr6g0311421	6	68.36	2	3	471	156	I
RcWRKY50	RchiOBHm_Chr7g0189781	7	9.05	1	2	501	166	I
RcWRKY51	RchiOBHm_Chr7g0195191	7	13.12	2	3	1080	359	III
RcWRKY52	RchiOBHm_Chr7g0196571	7	14.58	1	2	672	223	II
RcWRKY53	RchiOBHm_Chr7g0202671	7	20.25	2	3	879	292	II
RcWRKY54	RchiOBHm_Chr7g0223361	7	45.05	3	4	1374	457	II
RcWRKY55	RchiOBHm_Chr7g0241021	7	67	2	3	831	276	II
RcWRKY56	RchiOBHm_Chr7g0241041	7	67.01	4	5	429	142	II

^a^Available at https://lipm-browsers.toulouse.inra.fr/pub/RchiOBHm-V2/

^b^Chromosome

^c^Starting position (Mb)

### Phylogenetic analysis of the rose *WRKY* genes

**Fig. 1:**
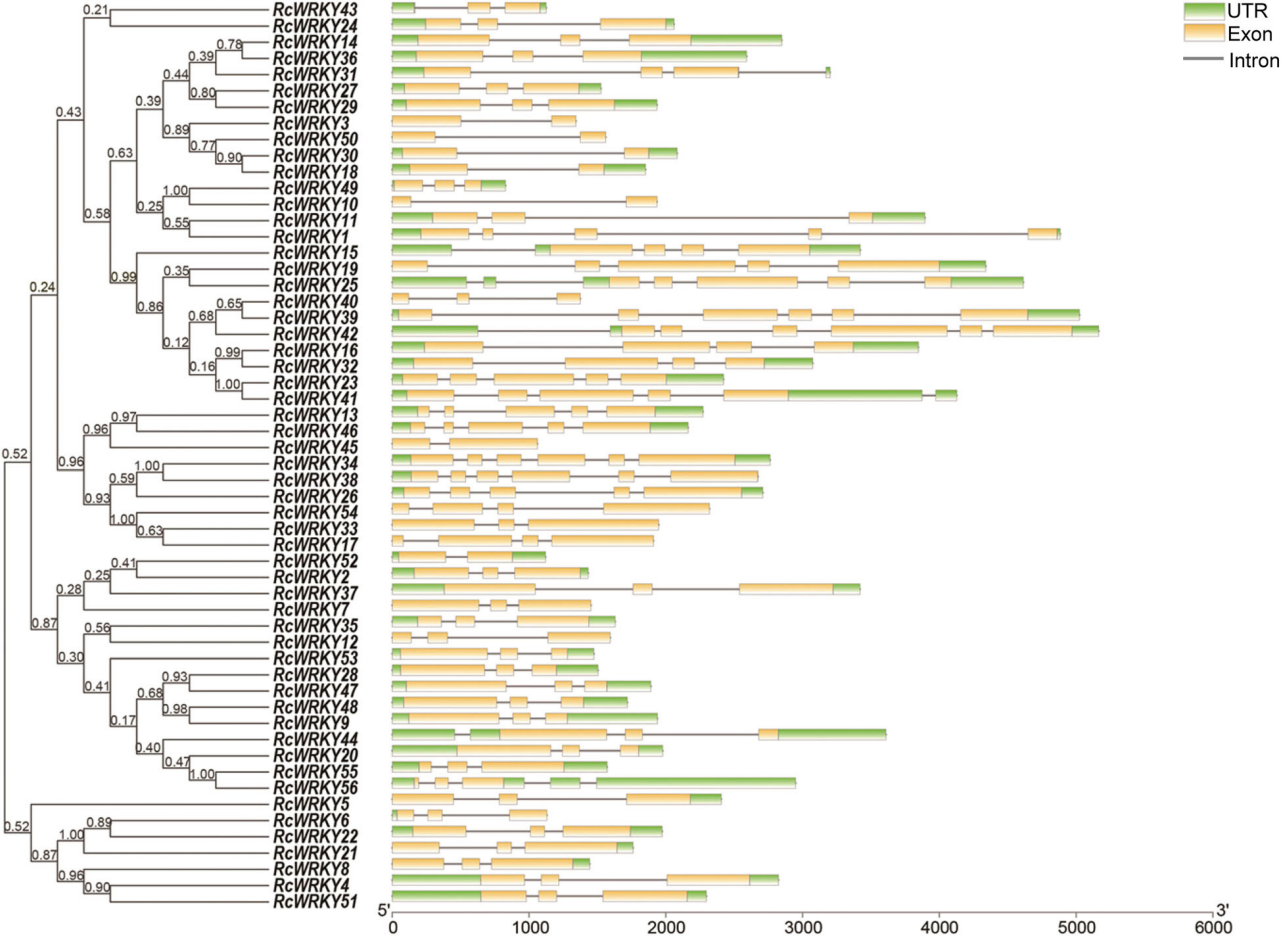
Phylogenetic analysis of the rose WRKY transcription factors. A complete alignment of the rose *WRKY*s was used to construct the phylogenetic tree

**Fig. 2:**
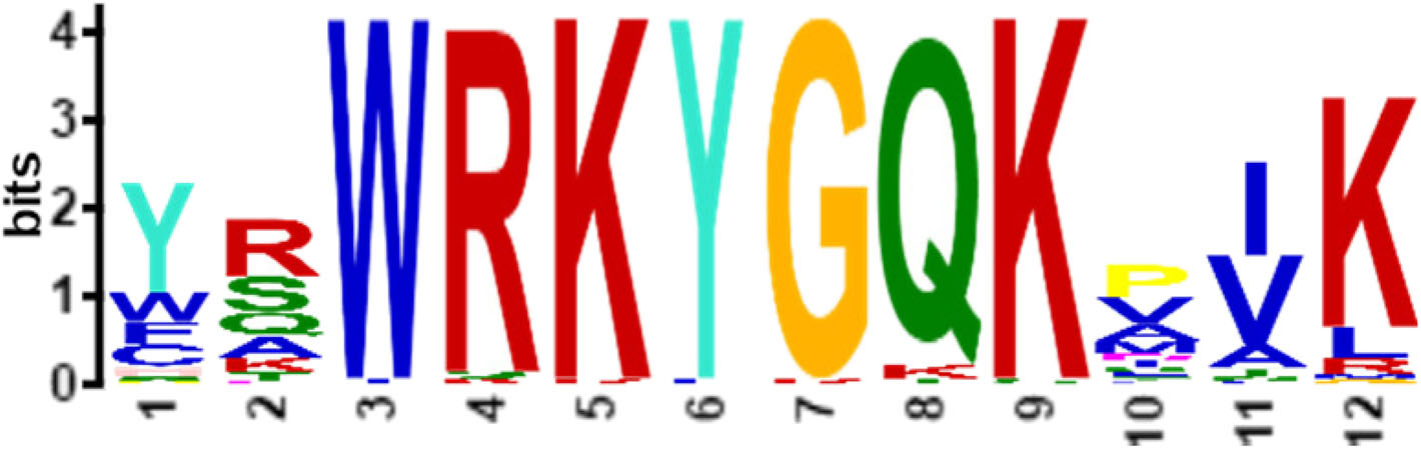
The sequence of the WRKY motif in rose WRKY proteins. These sequences were determined from the multiple alignment analysis of 56 RcWRKY transcription factors. The bit score indicates the informational content for each position in the sequence

A phylogenetic analysis of the *RcWRKY* genes was performed using the neighbor-joining method (Fig. 1). Our subsequent analysis of the exon-intron structures of the *RcWRKY*s was consistent with the results of the phylogenetic analysis. The *RcWRKY*s contain one to six introns, and most of the *RcWRKY* genes in the same clade exhibited similar exon-intron structures, such as *RcWRKY8*, *RcWRKY51*, *RcWRKY4*, *RcWRKY5*, *RcWRKY21*, *RcWRKY6*, and *RcWRKY22* (Table 1; Fig. 1). Some exceptions were also observed; for example, *RcWRKY39* and *RcWRKY40* were grouped into the same clade, but *RcWRKY40* has two introns and *RcWRKY39* has five. Moreover, the lengths of the *RcWRKY* introns are highly variable, ranging from tens to thousands of nucleotides. *RcWRKY11* contains the longest intron, comprising 2369 bp, while the shortest intron (51 bp) was found in *RcWRKY16*. In addition, we analyzed the conserved WRKY motif sequence in the rose WRKY proteins (Fig. 2).

**Fig. 3:**
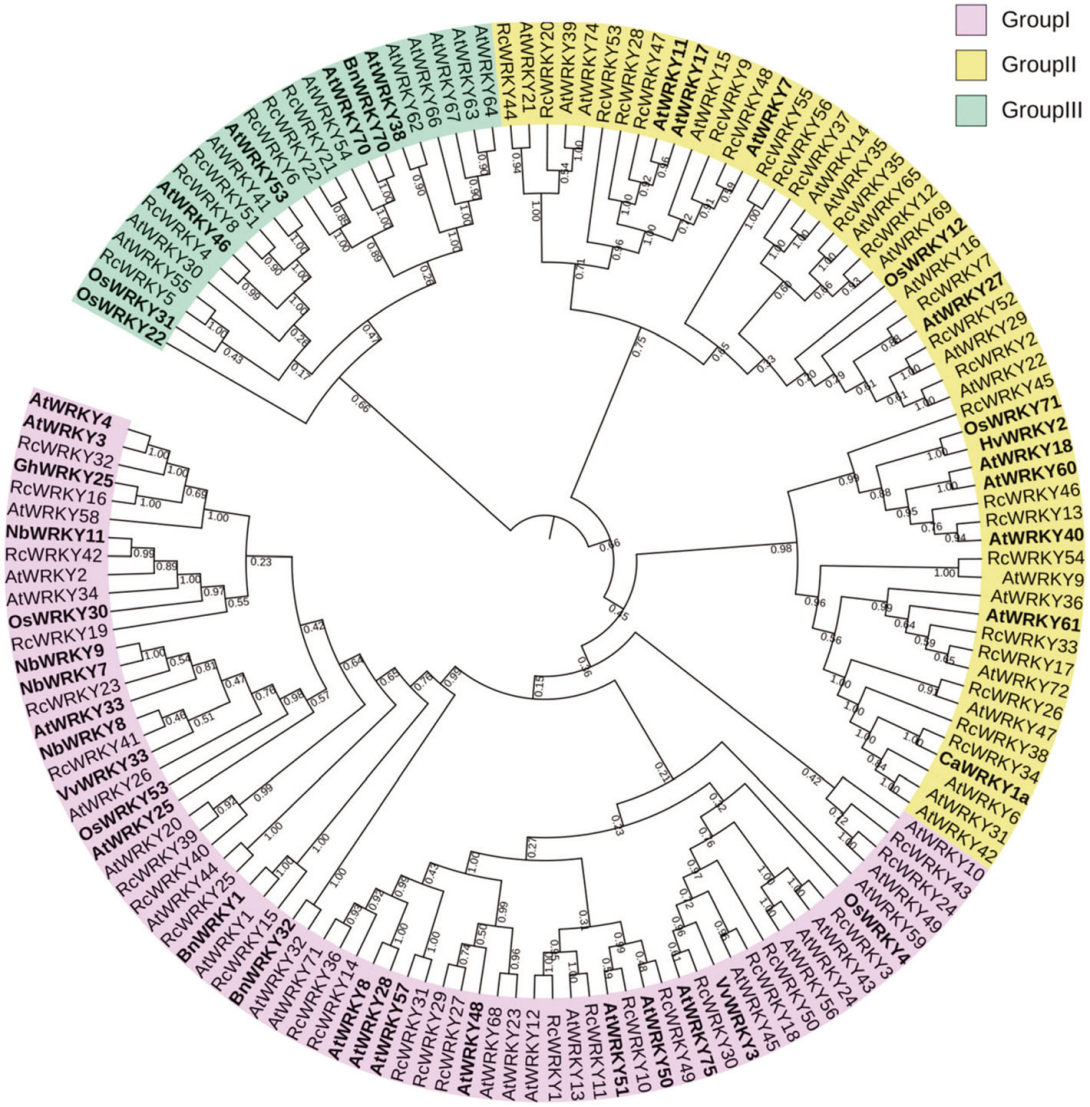
Phylogenetic analysis of the WRKY transcription factors in rose, Arabidopsis, and other plant species. Complete alignments of the rose and Arabidopsis WRKY sequences, and the disease-resistance-related WRKY transcription factors from a variety of plant species, including cotton (*Gossypium hirsutum*), rice (*Oryza sativa*), oilseed rape (*Brassica napus*), grape (*Vitis vinifera*), tobacco (*Nicotiana benthamiana*), barley (*Hordeum vulgare*), and pepper (*Capsicum annuum*), were generated to construct a phylogenetic tree using the Neighbor-Joining method. The bootstrap values are indicated on the nodes of the branches. The WRKYs reported to be involved in plant disease resistance are marked in bold

A total of 66 *AtWRKY* genes were previously identified in Arabidopsis [13]. There is also increasing evidence that the WRKY transcription factors play a key role in disease resistance in various plant species (Additional file 2: Table S1). To evaluate the evolutionary relationships among the RcWRKYs, AtWRKYs, and the plant WRKYs known to be involved in the regulation of disease resistance, we generated a composite phylogenetic tree using the neighbor joining method (Fig. 3). The AtWRKYs were previously divided into three groups, according to their evolutionary relationships, with those in Group I containing two WRKY domains and those in Groups II and III containing just one WRKY domain [13]. In the present study, we found that the evolutionary relationships of the *RcWRKY*s were consistent with the Arabidopsis WRKY Groups; the *RcWRKY* genes clustered with the Group I *AtWRKY* genes contained two WRKY domains, while the other *RcWRKY* genes contained just one WRKY domain. We found that the WRKYs reported to take part in the regulation of the disease response were distributed across all three Groups.

### Chromosomal locations, gene duplication, and Microsynteny

**Fig. 4:**
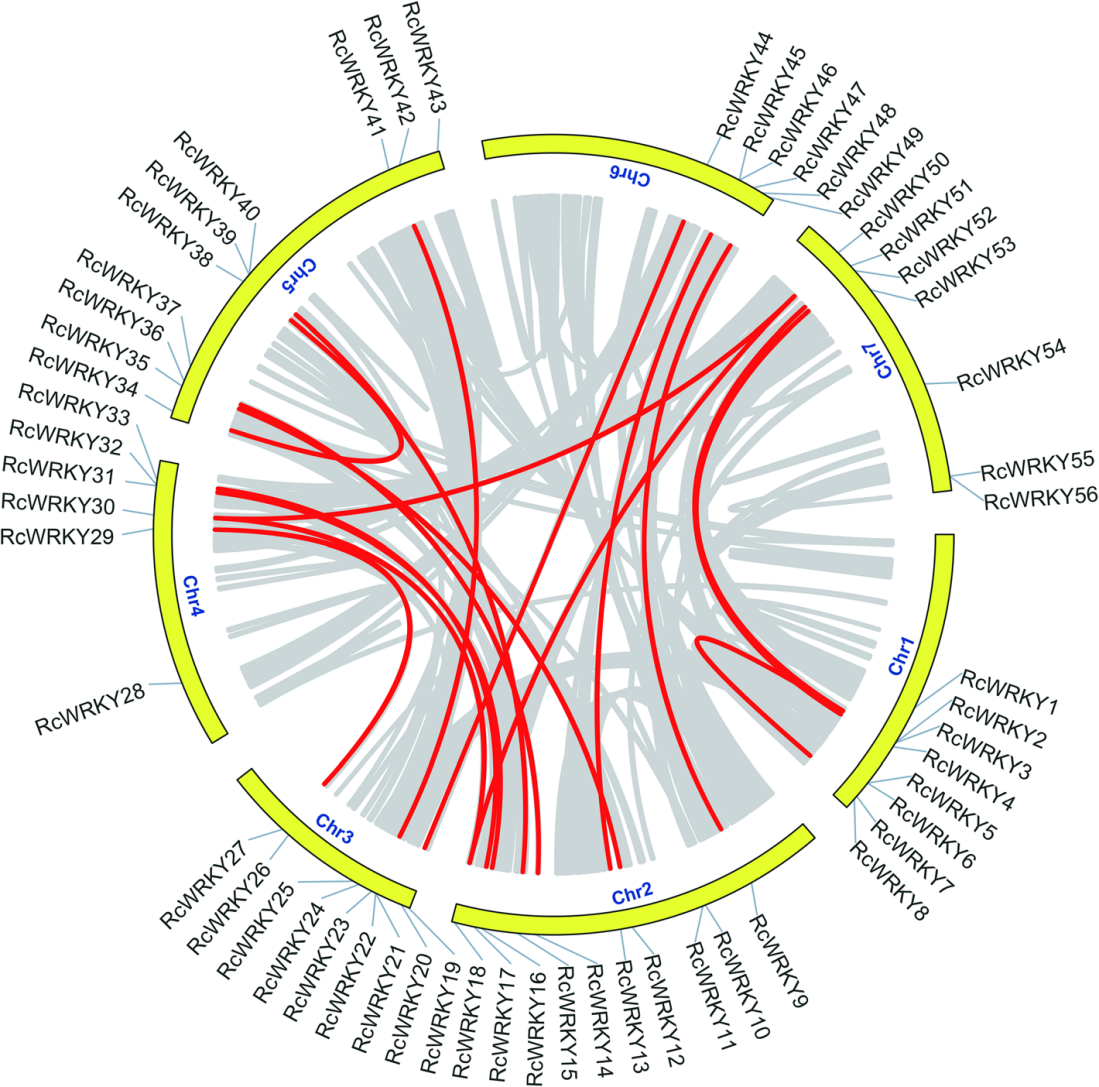
Localization and synteny of the *WRKY* transcription factors in the rose genome. The *RcWRKY*s were mapped to the rose chromosomes. Those with a syntenic relationship are joined by red lines. The gray lines indicate all syntenic blocks in the rose genome

The *RcWRKY* genes are unevenly distributed across all seven rose chromosomes (Table 1; Fig. 4; Additional file 1: Figure S1). We observed a high density of *RcWRKY*s in several regions, including the short arm of chromosome 3 and the long arm of chromosomes 1 and 6. In contrast, *RcWRKY* genes were not found on the short arm of chromosomes 1 and 6. Chromosomes 2 and 5 contain the largest numbers of *RcWRKY* genes (10), followed by chromosome 3 (9), while the lowest numbers of *RcWRKY* genes (6) were found on chromosomes 4 and 6. Chromosomes 1, 3, and 7 contain *RcWRKY* genes from all three Groups, whereas the other chromosomes only included *RcWRKY* genes from Groups I and II. The imbalance of the *RcWRKY* locations across the rose chromosomes implied that genetic variation occurred during evolution.

We further investigated the gene duplication events of the *RcWRKY*s. A total of 17 gene pairs were found in the rose genome (Table 2). Where two repeated genes are located on the same chromosome (*RcWRKY2*/*RcWRKY7* and *RcWRKY34*/*RhRKYY38*), they are likely to be tandem repeats. Other *RcWRKY* gene pairs are located on different chromosomes, suggesting that segmental duplications occurred within these regions, which may have arisen during full genome duplication in the roses [12]. The collinear relationship of the *RcWRKY* genes across the chromosomes is shown in Fig. 4.

**Table 2:** Duplication analysis of the *RcWRKY* gene family

Sequence1	Sequence2	Ka	Ks	Ka/Ks	Effective Len	Average S-sites	Average N-sites
RcWRKY2	RcWRKY7	0.5781	2.173169	0.266017	939	221.0833	717.9167
RcWRKY27	RcWRKY29	0.517668	NaN	NaN	948	217.4167	730.5833
RcWRKY18	RcWRKY30	0.360378	2.11817	0.170136	564	123.0833	440.9167
RcWRKY16	RcWRKY32	0.397724	1.996123	0.199248	1458	339.25	1118.75
RcWRKY17	RcWRKY33	0.47967	1.902171	0.25217	1359	316.5	1042.5
RcWRKY12	RcWRKY35	0.481472	NaN	NaN	699	164.25	534.75
RcWRKY14	RcWRKY36	0.459658	3.074976	0.149484	987	222.9167	764.0833
RcWRKY34	RcWRKY38	0.383131	2.014811	0.190157	1494	342.9167	1151.083
RcWRKY15	RcWRKY39	0.633703	1.385551	0.457365	1395	329.5	1065.5
RcWRKY23	RcWRKY41	0.356736	1.316333	0.271008	1500	339.3333	1160.667
RcWRKY20	RcWRKY44	0.309884	1.32433	0.233993	927	205.75	721.25
RcWRKY13	RcWRKY45	0.620285	NaN	NaN	741	168.9167	572.0833
RcWRKY9	RcWRKY48	0.331141	NaN	NaN	891	218	673
RcWRKY18	RcWRKY50	0.436328	2.474255	0.176347	492	105.3333	386.6667
RcWRKY30	RcWRKY50	0.369102	1.186943	0.310968	480	102.0833	377.9167
RcWRKY4	RcWRKY51	0.512715	2.346934	0.218462	1008	221.5833	786.4167
RcWRKY2	RcWRKY52	0.489159	1.471413	0.332442	654	150.75	503.25

In order to study the selective constraints among the duplicated *RcWRKY* genes, the Ka/Ks nucleotide substitution ratios were calculated for the 17 gene pairs (Table 2). Generally, a Ka/Ks ratio > 1 is consistent with positive selection, while Ka/Ks < 1 indicates a purifying selection. The Ka/Ks ratios of all 17 duplicated gene pairs were < 1 (Table 2), indicating that the duplicated *RcWRKY*s had undergone a purifying selection with limited functional divergence during their evolutionary history.

### Expression patterns of the *RcWRKY* genes in response to *B. cinerea*

There is increasing evidence to suggest that members of the *WRKY* family play key roles in plant defense responses against various pathogens. This involves the upregulation of *WRKY* expression upon pathogen infection. To study the *RcWRKY* responses to *B. cinerea*, we obtained RNA-seq transcriptomic data from rose petals exposed to this pathogen at 30 h post inoculation (hpi) and 48 hpi [10]. In rose petals, *B. cinerea* conidia germinate at 24 hpi, and the early response to infection is considered to occur at 30 dpi, as no visible disease lesions form by this point. The 48 hpi timepoint corresponds to the later response, when the lesions were starting to expand from the inoculation points [10].

The expression of 19 *RcWRKY* genes (*RcWRKY2*, *RcWRKY4*, *RcWRKY7*, *RcWRKY8*, *RcWRKY13*, *RcWRKY18*, *RcWRKY21*, *RcWRKY23*, *RcWRKY28*, *RcWRKY29*, *RcWRKY30*, *RcWRKY33*, *RcWRKY34*, *RcWRKY35*, *RcWRKY38*, *RcWRKY41*, *RcWRKY46*, *RcWRKY51*, and *RcWRKY54*) was significantly increased at 48 hpi with *B. cinerea*, suggesting they might be involved in rose resistance against this pathogen. Among these *B. cinerea*-induced *RcWRKY*s, the expression of seven *RcWRKY* genes was also significantly increased at 30 hpi. These results suggest these WRKYs might be specific regulators of the early stages of the defense response to *B. cinerea* (Table 3).

**Table 3:** Expression of the *RcWRKY* genes under *B. cinerea* infection^a^

Gene^b^	Accession number	Group	log_2_Ratio 30hpi	log_2_Ratio 48hpi
*RcWRKY2*	RchiOBHm_Chr1g0357671	II	–	2.908
*RcWRKY4*	RchiOBHm_Chr1g0359091	III	2.104	1.966
*RcWRKY7*	RchiOBHm_Chr1g0378621	II	–	2.269
*RcWRKY8*	RchiOBHm_Chr1g0380121	III	2.864	3.92
*RcWRKY13*	RchiOBHm_Chr2g0133001	II	–	5.947
*RcWRKY18*	RchiOBHm_Chr2g0175911	I	3.162	5.883
*RcWRKY21*	RchiOBHm_Chr3g0460351	III	–	6.323
*RcWRKY23*	RchiOBHm_Chr3g0461481	I	1.22	3.598
*RcWRKY28*	RchiOBHm_Chr4g0398741	II	–	2.188
*RcWRKY29*	RchiOBHm_Chr4g0425801	I	–	1.386
*RcWRKY30*	RchiOBHm_Chr4g0429851	I	2.319	4.448
*RcWRKY33*	RchiOBHm_Chr4g0440391	II	–	5.654
*RcWRKY34*	RchiOBHm_Chr5g0002561	II	–	3.618
*RcWRKY35*	RchiOBHm_Chr5g0011581	II	1.107	2.229
*RcWRKY38*	RchiOBHm_Chr5g0040801	II	–	4.919
*RcWRKY41*	RchiOBHm_Chr5g0071811	I	1.673	3.79
*RcWRKY46*	RchiOBHm_Chr6g0299501	II	1.092	2.166
*RcWRKY51*	RchiOBHm_Chr7g0195191	III	1.85	1.94
*RcWRKY54*	RchiOBHm_Chr7g0223361	II	–	1.962

^a^The log2 transformed expression profiles were obtained from the RNA-seq dataset [10]

^b^The *RcWRKY*s undergo duplicate events are marked in bold

**Fig. 5:**
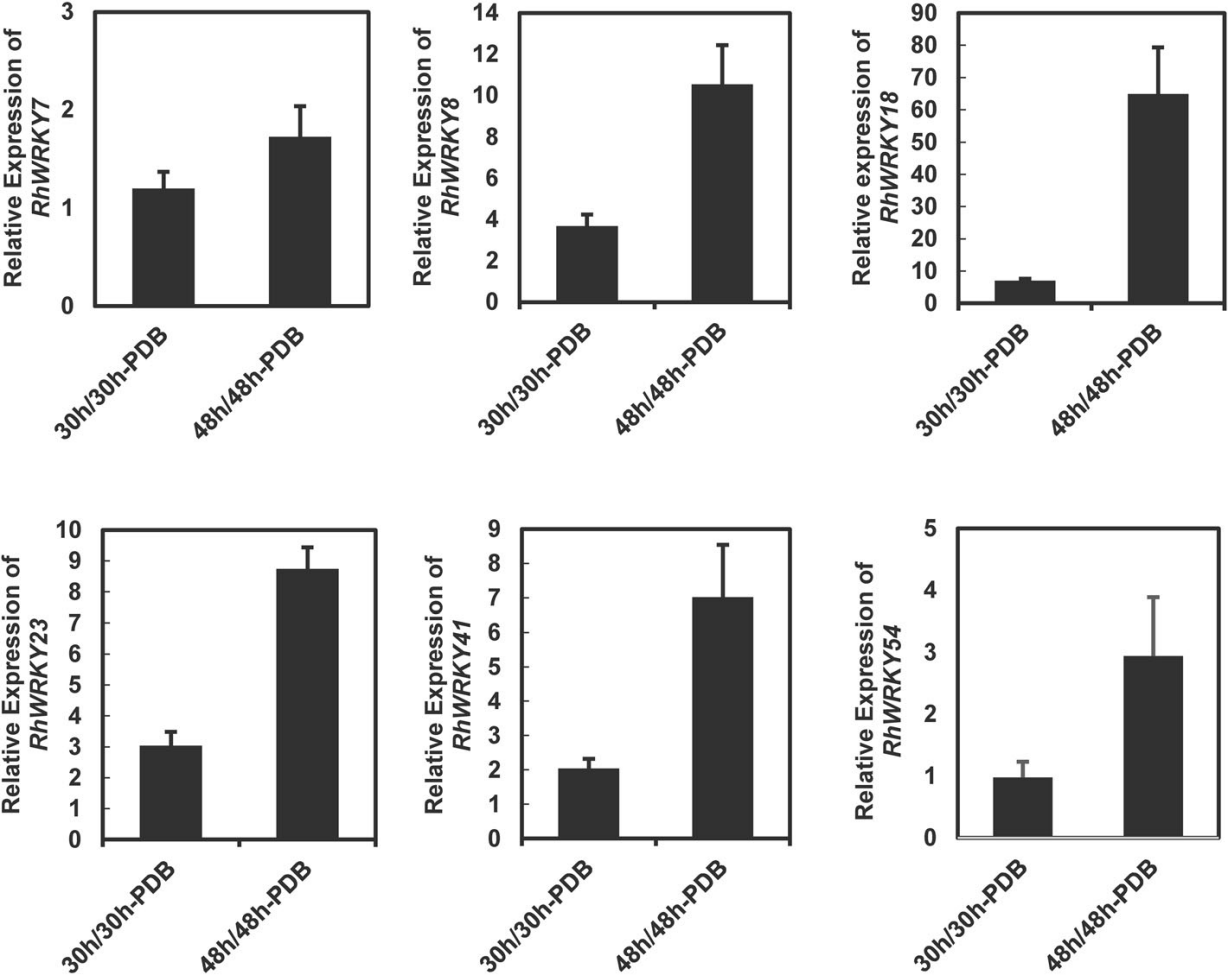
Validation of RNA-Seq results using qRT-PCR. RhUbi was used as an internal control. The primers used for determining transcript abundance are listed in Additional file 3: Table S2. PDB; potato dextrose broth; Values are the means of three biological replicates ± SD

To further validate the expression profiles from RNA-Seq, transcript abundance of six *RcWRKY* genes were analysis using qRT-PCR. The results from the qRT-PCR analysis were generally in agreement with the expression profiles obtained using the RNA-Seq data (Fig. 5).

### RcWRKY41 is required for rose resistance against *B. cinerea*

Using RNA-seq data taken from rose petals infected with *B. cinerea*, we identified 19 *B. cinerea*-inducible *WRKY* genes. To further illustrate the potential roles of these genes in the rose resistance against *B. cinerea*, we knocked down the expression of *RcWRKY41* using VIGS. *RcWRKY41* was selected for this VIGS study because 1) its expression is induced in both the early (30 hpi) and late (48 hpi) stages of *B. cinerea* infection (Fig. 5; Table 3), and it is therefore considered an important candidate regulator of resistance against this pathogen; and 2) RcWRKY41 belongs to Group I of the *RcWRKY*s, and is closely related to many *WRKY*s shown to play roles in disease resistance in various plant species, such as *NbWRKY7*, *NbWRKY8*, *NbWRKY9*,*VvWRKY33*, *OsWRKY53*, and *AtWRKY33* (Fig. 3; Additional file 2: Table S1).

**Fig. 6:**
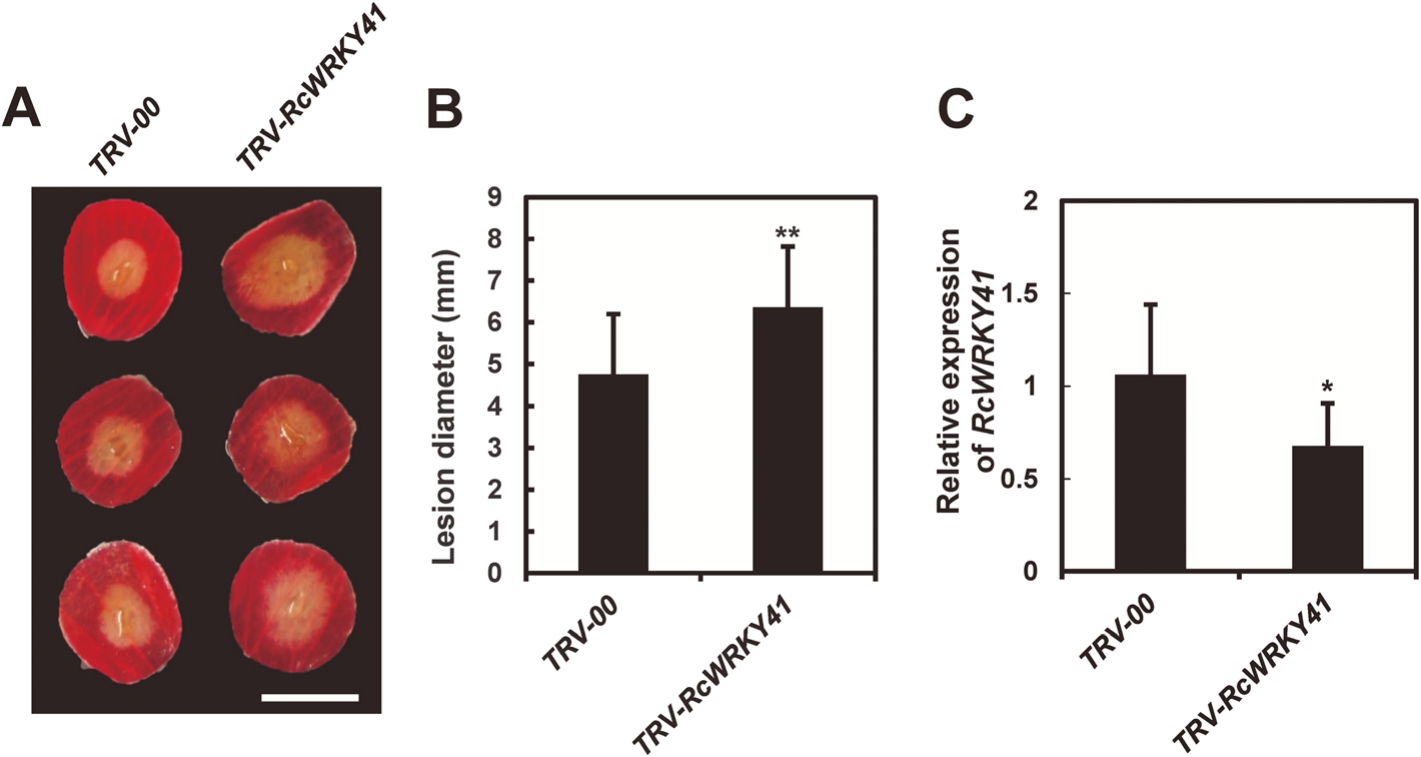
Functional analysis of rose transcription factor gene *RcWRKY41*. (A) Compromised *B. cinerea* resistance symptoms on rose petal disks upon the silencing of *RcWRKY41*, shown at 60 hpi (hours post inoculation). A recombinant tobacco rattle virus (TRV) targeting *RcWRKY41* (*TRV-RcWRKY41*) was used for the gene silencing, and an empty TRV (*TRV-00*) was used as the control. (B) Quantification of the average diameter of the disease lesions on the control and *RcWRKY41*-silenced petals at 60 hpi. Error bars = standard deviation. The statistical analysis was performed using a Student’s t-test; ** *P* < 0.01. (C) Quantification of *RcWRKY41* expression in TRV-RcWRKY41-inoculated petal discs relative to that in the control

To test whether *RcWRKY41* is involved in providing resistance against *B. cinerea*, we knocked down the expression of *RcWRKY41* in rose petals. To this end, we cloned a fragment of the *RcWRKY41* coding sequence into pTRV2 vector [14] to generate *TRV-RcWRKY41*. Agrobacterium cells carrying *TRV-RcWRKY41* and *TRV1* [14] constructs were mixed in a 1:1 ratio, then vacuum-infiltrated into the rose petal disks to generate *RcWRKY41*-silenced rose petals. The silenced petals were subsequently inoculated with *B. cinerea*. Compared with the control petals inoculated with the empty TRV vectors (*TRV-00*), plants inoculated with *TRV-RcWRKY41* showed more severe disease symptoms and their lesion sizes increased significantly (Fig. 6a and b). We further confirmed the silencing efficiency of VIGS by qRT-PCR (Fig. 6c). These results indicate that *RcWRKY41* plays an important role in the resistance of roses against *B. cinerea*.

## Discussion

Transcription factors often control a cluster of functionally related genes, and are therefore appropriate targets for the genetic engineering of (broad-spectrum) resistant crops. The *WRKY* genes are a major family of plant transcription factors with many important functions, including in the response to pathogens. Systematic and comprehensive genome-wide analyses of the WRKY family have previously been performed in Arabidopsis [13], rice [15], tomato [16], cotton (*Gossypium raimondii* and *G. hirsutum*) [17], cucumber (*Cucumis sativus*) [18], poplar (*Populus trichocarpa*) [19], and other species; however, a comprehensive analysis of the *RcWRKY* gene family has not previously been reported, leaving the functions of the rose WRKYs largely unclear. The rose (*R. chinensis*) genome sequencing project was recently completed, providing useful tools for the genome-wide analysis of the *RcWRKY* gene family. In this study, we comprehensively analyzed the *WRKY* family in rose, including their phylogeny, gene structures, chromosomal locations, gene duplication events, and expression profiles under *B. cinerea* infection.

The rose *RcWRKY* family contains more genes (56) than were reported in cucumber (55), but fewer than the number reported in Arabidopsis (66), rice (98), tomato (81), cotton (116 in *G. raimondii* and 102 in *G. hirsutum*), and poplar (104), indicating that the *WRKY* family expanded to varying degrees in different plant species following various gene duplication events during their evolution. Gene duplication was found to play a very important role in the expansion of this gene family in rose; a total of 17 duplication events were identified in the 56 *RcWRKYs*, the majority of which (15) involved segmental duplications, while two involved tandem duplications. The Ka/Ks ratios of all 17 *RcWRKY* pairs were < 1, indicating that this gene family has undergone purifying selection rather than positive selection, and suggested that the RcWRKYs were highly conserved. In plants, the *Resistance* (*R*) genes encoding the immune receptors that recognize a specific pathogen are often under positive selection pressure [20]. The purifying selection detected for all *RcWRKY*s therefore suggests they may be involved in the basal defense of plants, rather than in race-specific resistance.

Most of the clades identified in the phylogenetic analysis contained WRKYs from both Arabidopsis and rose, implying that these two species underwent fairly conservative evolution. There are some exceptions however; for example, AtWRKY38, AtWRKY62, AtWRKY63, AtWRKY64, AtWRKY66, and AtWRKY67 belong to an evolutionary clade that does not contain any RcWRKYs. This indicated that, after diverging from their common ancestor, these WRKY genes were either lost in rose or acquired (through duplication and divergence) in Arabidopsis.

Many *WRKY* genes have been shown to be involved in disease resistance in plants, prompting us to search for candidate *WRKY* genes involved in the rose response to *B. cinerea* infection. The elucidation of gene expression patterns often provides clues about their functions; therefore, we examined the expression changes in the *RcWRKY*s when exposed to *B. cinerea* infection. A total of 19 *RcWRKY* genes were found to be significantly upregulated upon *B. cinerea* infection in rose petals, most of which (14 of 19) had undergone gene duplication events. We further identified the *RcWRKY*s that might participate in *B. cinerea* resistance by adding them to a phylogenetic tree of the plant *WRKY*s known to be involved in the disease responses. Among the 19 *B. cinerea*-induced *RcWRKY*s, *RcWRKY41* was shown to be evolutionally close to a number of disease resistance *WRKY*s from various plant species, and its expression was found to increase from the early to late stages of the *B. cinerea* infection. *RcWRKY41* was therefore considered to be a candidate gene participating in *B. cinerea* resistance, which was confirmed when VIGS was used to silence its expression in rose petals, resulting in their reduced resistance to *B. cinerea.* This indicates *RcWRKY41* plays an important positive regulatory function in the resistance of rose petals against grey mold.

## Conclusions

We performed a genome-wide analysis of the *RcWRKY*s, exploring their phylogenetic relationships, collinearity, and expression profiles. A total of 56 non-redundant rose *RcWRKY* family members were identified, which could be divided into three groups based on our analyses of their phylogeny and conserved domains; 22 of them belonged to Group I, 26 belonged to Group II, and nine belonged to Group III. Our expression analysis indicated that 19 *RcWRKY* family genes were induced in rose petals subjected to a *B. cinerea* infection. By comparing these sequences with other plant WRKYs known to be involved in disease resistance, we revealed that *RcWRKY41* is involved in the regulation of gray mold resistance in rose petals, which was confirmed using VIGS. These results provide new information that may facilitate the further functional analysis of the *RcWRKY*s in roses.

## Methods

### Identification and characteristics of the *WRKY* genes in the rose genome

The complete rose (*Rosa chinensis* ‘Old Blush’) genome sequence was obtained from https://lipm-browsers.toulouse.inra.fr/pub/RchiOBHm-V2/. To identify the non-redundant *WRKY* genes in the rose genome, the consensus protein sequence of the WRKY Hidden Markov Model (HMM) was downloaded from Pfam (PF03106; http://pfam.xfam.org). This HMM profile was then used as a query to search the rose genome, resulting in the identification of all rose sequences containing a WRKY domain with an E-value <1e^− 3^. Finally, all candidate RcWRKYs were validated using the Pfam and the Conserved Domains Database (CDD; https://www.ncbi.nlm.nih.gov / Structure / cdd / wrpsb.cgi) to determine that they contained the core domains.

### Phylogenetic analyses

A total of 66 Arabidopsis WRKY protein sequences were collected from The Arabidopsis Information Resource (TAIR) (http://www.arabidopsis.org/). Based on the results of previous studies, additional sequences of *WRKY* genes involved in plant disease resistance were collected from GenBank, including those from cotton (*Gossypium hirsutum*), rice (*Oryza sativa*), oilseed rape (*Brassica napus*), grape (*Vitis vinifera*), tobacco (*Nicotiana benthamiana*), barley (*Hordeum vulgare*), and pepper (*Capsicum annuum*). A phylogenetic analysis was used to determine whether orthologs of these genes are present in the rose genome. The amino acid sequences of WRKY proteins were aligned using ClustalW. The alignment of WRKY sequences was used to perform the phylogenetic analysis. Phylogenetic dendrograms were constructed using the neighbor-joining (NJ) method in MEGA 6.0 software [21]. The percentage of replicate trees in which the associated taxa clustered together in the bootstrap test (1000 replicates) are shown next to the branches. The evolutionary distances were computed using the p-distance method and are in the units of the number of amino acid differences per site. All positions with less than 50% site coverage were eliminated.

### Collinearity analyses

In order to identify collinearity, a Multiple Collinearity Scan toolkit [22] was used to detect the microsyntenic relationships between the *WRKY* genes. The resulting microsynteny chains were then evaluated using ColinearScan (E-value <1e^− 10^).

### Calculation of the non-synonymous (Ka) to synonymous (Ks) nucleotide substitution ratio

An analysis of the Ka/Ks ratios was used to determine the selection modes driving the evolution of the *RcWRKY*s. These ratios were calculated using TBtools software [23].

### Expression of the *RcWRKY*s in response to *B. cinerea*

RNA-Seq data from rose petals infected with *B. cinerea* were obtained from the National Center for Biotechnology Information (NCBI) database (accession number PRJNA414570). Clean sequencing reads were mapped to the rose reference genome, and the number of reads per kb per million reads (RPKM) were used to determine the gene expression levels. To confirm the RNA-Seq results, the transcript abundance of 6 *RcWRKY* genes was analyzed using qRT-PCR. To this end, cDNA was generated from rose petals inoculated with *B. cinerea*, using Takara Reverse Transcriptase M-MLV (Takara). Quantitative RT-PCR was performed on a StepOnePlus Real-Time PCR System (Thermo Fisher Scientific), by using 1 μL of the first strand cDNA in the reaction with the KAPA SYBR rapid quantitative PCR kit (KAPA Biosystems). *RhUbi* was used as a housekeeping gene. The primers used for determining transcript abundance are listed in Additional file 3: Table S2.

### VIGS

To obtain the *TRV-RcWRKY41* construct, a fragment from the coding region of *RcWRKY41* was amplified using the primer pairs RcWRKY41-TRV-F (5′-GGGGACAAGTTTGTACAAAAAAGCAGGCTTTTACCAAGCCACAATACCAA-3′) and RcWRKY41-TRV-R (5′-GGGGACCACTTTGTACAAGAAAGCTGGGTAACACAGCAATGATTCAAAA-3′) and cloned into the *Tobacco rattle virus* vector *TRV2* [14]. To establish VIGS in rose petals, petals were detached from the outermost whorls of rose flowers (*R. hybrida* ‘Samantha’) during stage 2 of flower opening. A 15-mm disk was then punched from the center of each petal. *Agrobacterium tumefaciens* cultures containing constructs expressing *TRV1* [14] and recombinant *TRV2* were mixed in a 1:1 ratio and vacuum-infiltrated into the petal disks. At 6 days after the TRV infection, the petal disks were inoculated with *B. cinerea*. The VIGS was repeated at least three times using at least 48 disks. After the *B. cinerea* inoculation, the lesion sizes were recorded, and a Student’s *t*-test was conducted to identify any significant differences.

## Supplementary information

**Additional file 1: Figure S1.** Chromosomal distribution of the *RcWRKY* genes. The physical location of each *RcWRKY* gene is listed on the left side of the chromosomes.

**Additional file 2: Table S1.** Plant *WRKY* family genes involved in disease resistance [8, 24–54].

**Additional file 3: Table S2.** List of primers used in this study.

## Data Availability

The datasets used and/or analyzed during the current study has been included within supplemental data. The plant materials are available from the corresponding author on reasonable request.

## Abbreviations

CDD: Conserved Domains Database
HMM: Hidden Markov Model
Hpi: Hours post inoculation
NJ: Neighbor-joining
RPKM: Number of reads per kb per million reads
VIGS: Virus-induced gene silencing
