# Supplementary figures and images for: Genome-wide characterization of the rose (Rosa chinensis) WRKY family and role of RcWRKY41 in gray mold resistance
Source: BMC Plant Biol. 2019 Nov 27;19:522. doi: 10.1186/s12870-019-2139-6 (PMC6882016; doi:10.1186/s12870-019-2139-6)

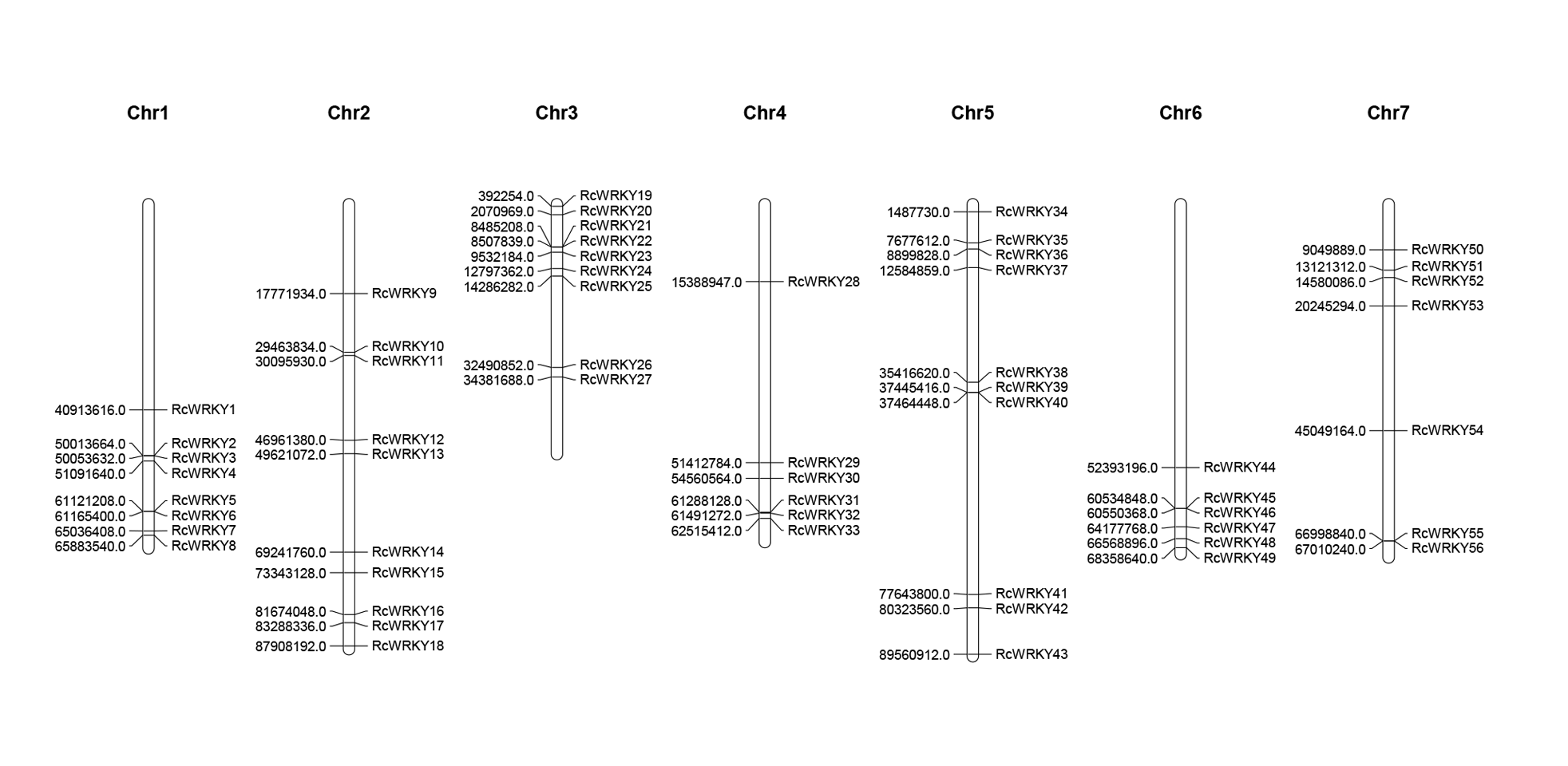

Supplement: Supplementary file 1 — Additional file 1: Figure S1. Chromosomal distribution of the RcWRKY genes. The physical location of each RcWRKY gene is listed on the left side of the chromosomes. [file 12870_2019_2139_MOESM1_ESM.tif]
