# Supplementary material for: Genome-wide characterization of the rose (Rosa chinensis) WRKY family and role of RcWRKY41 in gray mold resistance
Source: BMC Plant Biol. 2019 Nov 27;19:522. doi: 10.1186/s12870-019-2139-6 (PMC6882016; doi:10.1186/s12870-019-2139-6)
Supplement: Supplementary file 2 — Additional file 2: Table S1. Plant WRKY family genes involved in disease resistance [8, 24–54]. [file 12870_2019_2139_MOESM2_ESM.docx]

**Supplemental Table S1 Plant *WRKY* family genes involved in disease resistance**

| **Gene name** | **Gene ID** | **Species** | **Pathogens** | **References** |
| --- | --- | --- | --- | --- |
| AtWRKY3 | At2g03340 | [*Arabidopsis thaliana*](javascript:;) | *Botrytis cinerea* | [24] |
| AtWRKY4 | At1g13960 | [*Arabidopsis thaliana*](javascript:;) | *Botrytis cinerea*  *Pseudomonas syringae* | [24] |
| AtWRKY7 | At4g24240 | [*Arabidopsis thaliana*](javascript:;) | *Pseudomonas syringae* | [25] |
| AtWRKY8 | At5g46350 | [*Arabidopsis thaliana*](javascript:;) | *Botrytis cinerea* | [26] |
|  |  |  | Tobacco mosaic virus |  |
| AtWRKY11 | At4g31550 | [*Arabidopsis thaliana*](javascript:;) | *Pseudomonas syringae* | [27] |
| AtWRKY17 | At2g24570 | [*Arabidopsis thaliana*](javascript:;) | *Pseudomonas syringae* | [28] |
| AtWRKY18 | At4g31800 | [*Arabidopsis thaliana*](javascript:;) | *Botrytis cinerea* | [29] |
|  |  |  | *Pseudomonas syringae* |  |
| AtWRKY25 | At2g30250 | [*Arabidopsis thaliana*](javascript:;) | *Pseudomonas syringae* | [30] |
| AtWRKY27 | At5g52380 | [*Arabidopsis thaliana*](javascript:;) | *Ralstonia solanacearum* | [31] |
| AtWRKY28 | At4g18170 | [*Arabidopsis thaliana*](javascript:;) | *Botrytis cinerea* | [32] |
|  |  |  | *Sclerotinia sclerotiorum* |  |
| AtWRKY33 | At2g38470 | [*Arabidopsis thaliana*](javascript:;) | *Botrytis cinerea* | [33] |
|  |  |  | *Alternaria brassicicola* |  |
|  |  |  | *Pseudomonas syringae* |  |
| AtWRKY38 | At5g22570 | [*Arabidopsis thaliana*](javascript:;) | *Pseudomonas syringae* | [34] |
| AtWRKY40 | At1g80840 | [*Arabidopsis thaliana*](javascript:;) | *Botrytis cinerea* | [29] |
| AtWRKY46 | At2g46400 | [*Arabidopsis thaliana*](javascript:;) | *Pseudomonas syringae* | [35] |
| AtWRKY48 | At5g49520 | [*Arabidopsis thaliana*](javascript:;) | *Pseudomonas syringae* | [36] |
| AtWRKY50 | At5g26170 | [*Arabidopsis thaliana*](javascript:;) | *Botrytis cinerea* | [37] |
| AtWRKY51 | At5g64810 | [*Arabidopsis thaliana*](javascript:;) | *Botrytis cinerea* | [37] |
| AtWRKY53 | At4g23810 | [*Arabidopsis thaliana*](javascript:;) | *Pseudomonas syringae* | [38] |
| AtWRKY57 | At1g69310 | [*Arabidopsis thaliana*](javascript:;) | *Botrytis cinerea* | [39] |
| AtWRKY60 | At2g25000 | [*Arabidopsis thaliana*](javascript:;) | *Botrytis cinerea* | [29] |
| AtWRKY61 | At1g18860 | [*Arabidopsis thaliana*](javascript:;) | *Turnip crinkle* virus | [40] |
| AtWRKY70 | At3g56400 | [*Arabidopsis thaliana*](javascript:;) | *Pseudomonas syringae* | [27] |
| AtWRKY75 | At5g13080 | [*Arabidopsis thaliana*](javascript:;) | *Sclerotinia sclerotiorum* | [32] |
|  |  |  | *Pseudomonas syringae* |  |
| GhWRKY25 | JF899343 | *Gossypium hirsutum* | *Botrytis cinerea* | [41] |
| VlWRKY3 | XM_002275540.3 | *Vitis labrusca×V. vinifera* | *Botrytis cinerea* | [42] |
| VvWRKY33 | XP_002264974.1 | *Vitis vinifera* | *Plasmopara viticola* | [43] |
| NbWRKY7 | AB445391.1 | *Nicotiana benthamiana* | *Botrytis cinerea* | [44] |
| NbWRKY8 | AB445392.1 | *Nicotiana benthamiana* | *Botrytis cinerea* | [44] |
| NbWRKY9 | AB711130 | *Nicotiana benthamiana* | *Botrytis cinerea* | [44] |
| NbWRKY11 | AB711132 | *Nicotiana benthamiana* | *Botrytis cinerea* | [44] |
| OsWRKY30 | Os08g0499300 | [*Oryza sativa*](javascript:;) | *Rhizoctonia solani* | [45, 46] |
| OsWRKY53 | Os05g0343400 | [*Oryza sativa*](javascript:;) | *Magnaporthe oryzae* | [47] |
| OsWRKY12 | Os01g0624700 | [*Oryza sativa*](javascript:;) | *Xanthomonas oryzae pv. oryzae* | [48] |
| OsWRKY4 | Os03g0758900 | [*Oryza sativa*](javascript:;) | *Rhizoctonia solani* | [49] |
| OsWRKY22 | Os01g0820400 | [Oryza sativa](javascript:;) | *Magnaporthe oryzae* | [50] |
| OsWRKY71 | Os02g0181300 | [Oryza sativa](javascript:;) | *Xanthomonas oryzae pv. oryzae* | [51] |
| OsWRKY31 | Os03g0321700 | [*Oryza sativa*](javascript:;) | *Magnaporthe oryzae* | [52] |
| HvWRKY2 | Aj858838 | *Hordeum vulgare* | *Blumeria graminis hordei* | [53] |
|  |  |  | *Melampsora lini* |  |
| BnWRKY1 | EU912389 | [*Brassica napus*](http://cpfd.cnki.com.cn/Article/CPFDTOTAL-CSSC201811003053.htm) | *Sclerotinia sclerotiorum* | [54] |
| BnWRKY32 | EU912402 | [*Brassica napus*](http://cpfd.cnki.com.cn/Article/CPFDTOTAL-CSSC201811003053.htm) | *Sclerotinia sclerotiorum* | [54] |
| BnWRKY70 | EU912415 | [*Brassica napus*](http://cpfd.cnki.com.cn/Article/CPFDTOTAL-CSSC201811003053.htm) | *Sclerotinia sclerotiorum* | [54] |
| CaWRKY1a | DQ335599 | *Capsicum annuum* | *Xanthomonas axonopodis* pv. *glycines* | [55] |
