# Supplementary material for: Genome-wide characterization of the rose (Rosa chinensis) WRKY family and role of RcWRKY41 in gray mold resistance
Source: BMC Plant Biol. 2019 Nov 27;19:522. doi: 10.1186/s12870-019-2139-6 (PMC6882016; doi:10.1186/s12870-019-2139-6)
Supplement: Supplementary file 3 — Additional file 3: Table S2. List of primers used in this study. [file 12870_2019_2139_MOESM3_ESM.docx]

| Gene name | Primer sequence（5’-3’） |
| --- | --- |
| RcWRKY7 | F：GAGGAAGCCGAGGATGAGGAAAT |
|  | R：ACCAGAAGTTCCAGCAGCCAAAGA |
| RcWRKY8 | F：ATTATGCGGGGAACTTTTCTCCTTC |
|  | R：TGGTTACCACCAAAGTCCTGCTGAG |
| RcWRKY18 | F：GTCAAAGCCAACAAATTCCCCAGAA |
|  | R：CTTCGTAAGTGGTCACCACAATGCC |
| RcWRKY23 | F：GGAGCGAGCATCTCACGATACGA |
|  | R：GCTGTTGGCATCAGAAGCAGGTCTA |
| RcWRKY41 | F：TGACAATCTAAGGCTACCGACATCA |
|  | R：TTATCAAACCCTGCAAATCCATAAC |
| RcWRKY54 | F：GGCAAACCTGCAAATTATCACCATA |
|  | R：CCCAGATGTCCTCCTGCTGTTACTC |
| RcUBI2 | F：GCCCTGGTGCGTTCCCAACTG |
|  | R：CCTGCGTGTCTGTCCGCATTG |

**Supplemental Table S2.** List of primers used in this study
